# Genotype distribution and molecular characterization of HPV in the Peruvian amazon: insights into prevalence, lineage diversity, and viral integration

**DOI:** 10.1038/s41598-025-18455-3

**Published:** 2025-09-15

**Authors:** Lesly Solis-Ponce, Milan Stosic, Greisi Curicó, Susan Espetia, Heidy Sanchez-Grandez, Sadaf Sakina Hassan, Jorge Basiletti, Kristiane Søreng, Maribel Acuña-Barrios, Lilian Huarca-Balbin, Mariela Yaya-Ríos, Giovanny Vilcarino-Zevallos, Renzo Lopez, Andrea Matos, Cesar Ramal-Asayag, George Obregon, Laila Sara Arroyo Mühr

**Affiliations:** 1 Peruvian HPV National Reference Laboratory. National Reference Laboratory for Sexually Transmitted Viruses, National Institute of Health, Avenida Defensores del Morro 2268, 15067 Chorrillos, Lima Peru; 2 Norwegian HPV Reference Laboratory, Department of Microbiology and Infection Control, Akershus University Hospital, 1478 Lørenskog, Norway; 3 Infectious and Tropical Diseases Research Laboratory, Loreto Regional Hospital, Avenida 28 de Julio S/N , 16004 Loreto, Maynas Peru; 4 International HPV Reference Center, Center for Cervical Cancer Elimination, F56, Karolinska University Hospital Huddinge, Karolinska Institutet, 141 86 Huddinge, Sweden; 5 Argentinian HPV Reference Laboratory, National Institute of Infectious Diseases-ANLIS “Dr. Malbrán”, C1282AFF Buenos Aires, Argentina; 6 Center for Evaluation of Health Technologies, National Institute of Health (Peru), Avenida Defensores del Morro 2268, 15067 Chorrillos, Lima Peru; 7 Loreto Regional Hospital, Avenida 28 de Julio S/N, 16004 Loreto, Maynas Peru; 8 Dirección de Prevención y Control del Cáncer, Ministerio de Salud, Av. Salaverry 801, 15072 Jesús María, Lima Peru; 9 School of Medicine, National University of the Peruvian Amazon, Av. Grau 1072, 16001 Iquitos, Peru

**Keywords:** Cervical cancer, Human papillomavirus, Sublineages, Integration, Peruvian amazon, TaME-seq, Cancer, Diseases, Microbiology, Oncology

## Abstract

Cervical cancer remains a leading cause of cancer-related mortality among women in Peru, with the highest death rates reported in the Amazon region. Despite this burden, molecular data on human papillomavirus (HPV) in this area are limited. This study aimed to assess HPV genotype distribution and molecular characteristics in women undergoing cervical screening in the Peruvian Amazon. Between May and November 2024, cervical samples were collected from 503 women aged 25–55, along with an additional subset of 35 women with histologically confirmed CIN2 + lesions. HPV genotyping was performed using the Allplex™ HPV28 assay, and single infections with HPV16 or HPV52 were further analyzed by TaME-seq to determine lineage, intrahost variability, and integration. HPV prevalence was 51.7% (278/538), with high rates of multiple infections (44.2%) and low vaccination coverage (7.8%). Oncogenic HPV types were detected in 35.7% (192/538) of participants, with HPV 16 and HPV 52 being the most prevalent. Sublineage analysis revealed predominance of A1 variants, while viral integration was detected in nine cases, three associated with CIN2 + lesions. Integration sites occurred mainly in *E1/E2* for HPV16 and *L1/L2* for HPV52. These findings highlight the urgent need to expand vaccination and implement molecular screening strategies in the Peruvian Amazon.

**Supplementary Information:**

The online version contains supplementary material available at 10.1038/s41598-025-18455-3.

## Introduction

Cervical cancer remains a major public health challenge globally, particularly in low- and middle-income countries and rural areas, where access to early detection and treatment remains limited^1^. In Peru, it is one of the leading causes of cancer-related mortality among women, primarily driven by persistent infection with high-risk human papillomavirus (HPV) genotypes. Although national efforts to expand HPV vaccination and cervical screening coverage have yielded progress, significant gaps persist, especially in remote and low-resource areas of the country.^2^.

Several studies have assessed HPV prevalence and genotype distribution in Peru. However, most have focused on urban or more accessible populations^3–5^. In contrast, limited molecular epidemiological data are available from the Peruvian Amazon, where health infrastructure is weaker, healthcare access is restricted, and cervical cancer outcomes are among the poorest in the country. The Peruvian Amazon, comprising regions such as Loreto, Ucayali, and Madre de Dios, reports some of the highest cervical cancer mortality rates in the country, exceeding 35 deaths per 100,000 women annually^1,6^.

To address these inequities, there is an urgent need to better understand the distribution of HPV genotypes and the molecular characteristics of circulating viral variants in these populations. While HPV16 and HPV18 are the most carcinogenic types globally, other genotypes, such as HPV52, have emerged as important contributors to cervical cancer risk in Latin America^3,7^. Understanding this genotype distribution is critical for tailoring screening strategies and informing vaccine policy.

This study represents the first comprehensive effort to characterize HPV genotype distribution and molecular features in a remote, low-resource population from the Peruvian Amazon. The investigation assessed the prevalence of HPV types and conducted molecular characterization of most predominant genotypes, including lineage assignment, viral integration, and intrahost variability. The objective was to generate insights into the diversity and molecular behavior of high-risk HPV types in this region, contributing to future risk stratification strategies and supporting the development of more effective, locally adapted screening and prevention programs.

## Results

### Study population characteristics

From May to November 2024, a total of 503 women aged 25–55 years consented to participate in the study. The median age was 39 years (range 25–55). HPV was detected in 251/503 participants (49.90%), of whom 113/251 (45.02%) exhibited multiple HPV infections. (NOTE: Multiple infections included combinations of different risk levels, with high-/low-risk, high-/medium-risk, and medium-/low-risk genotypes detected). Only 42/503 women (8.35%) reported prior HPV vaccination. A summary of the number of participants, age range, HPV status (positive/negative), infection pattern (single or multiple HPV types), and self-reported HPV vaccination status is presented in Table 1. Cytological results were not available for these women, as the protocol focused exclusively on HPV testing.

**Table 1:** Characteristics of women attending cervical screening clinics in loreto, Peru (May–November 2024).

Age	*n*	HPV negative	HPV positive	Single infection	Multiple infection	Non vaccinated	Vaccinated
25–30	97	35	62	29	33	77	20
31–35	94	54	40	23	17	86	8
36–40	108	58	50	26	24	102	6
41–45	77	39	38	21	17	74	3
46–50	86	45	41	30	11	84	2
51–55	41	21	20	9	11	38	3
Total	503	252	251	138	113	461	42

The table shows the distribution of participants by age group, HPV status (positive or negative), infection pattern (single or multiple HPV types), and self-reported HPV vaccination status. HPV, human papillomavirus.

An additional subset of 35 women with histologically confirmed CIN2 + lesions was recruited from pathology units during the same period (May–November 2024). The median age of this group was 41 years (range 29–55) and none of the participants in this subset had been vaccinated against HPV. Among them, 8/35 (22.86%) were HPV negative, 17/35 (48.57%) had a single HPV infection and 10/35 (28.57%) showed multiple HPV infections. Follow-up biopsies were confirmed (histologically) as CIN2 + lesions.

### HPV distribution

**Fig. 1:**
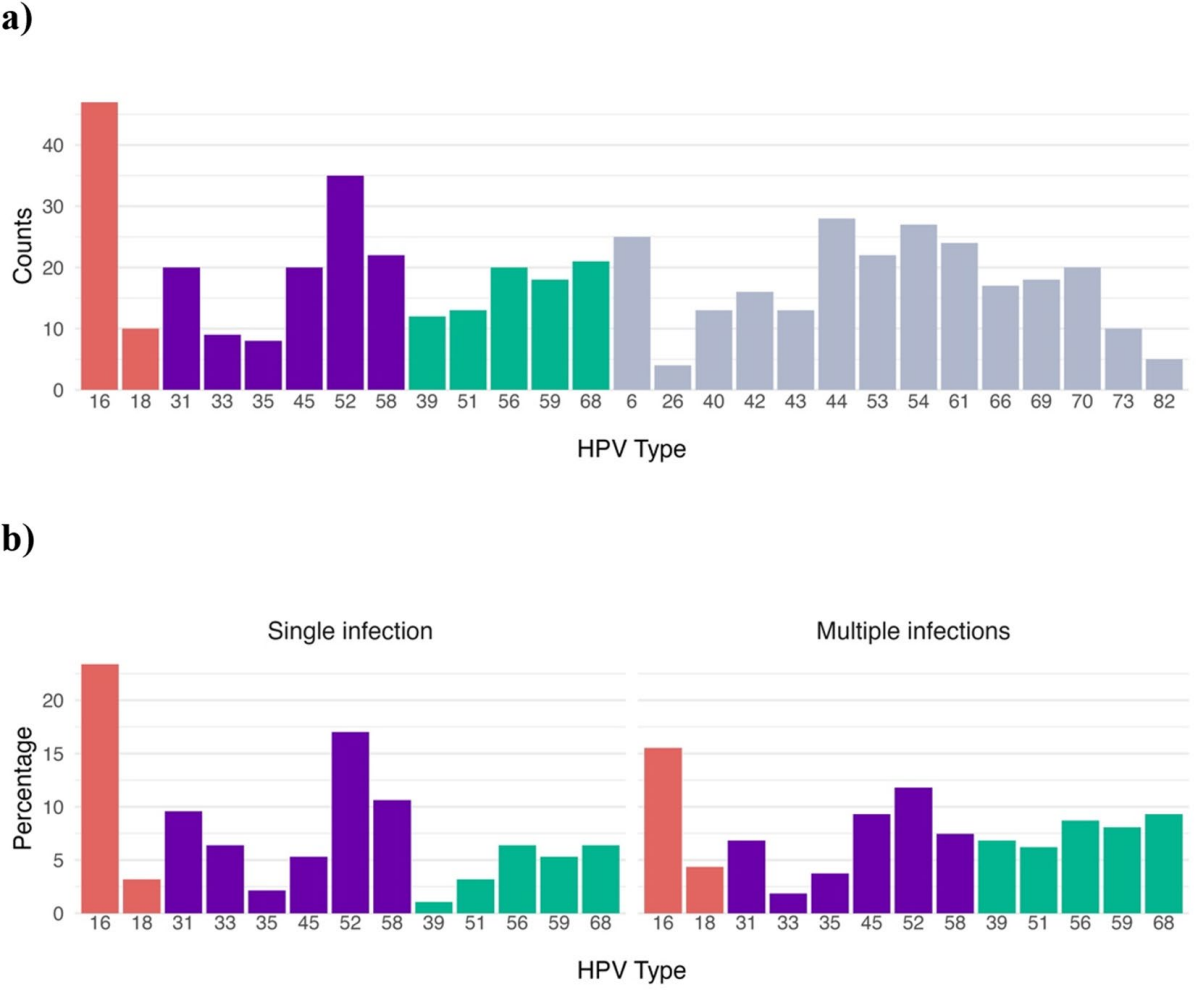
Distribution of HPV genotypes and oncogenic risk in cervical samples from the Peruvian Amazon. (**A**) Number of HPV detections by genotype in cervical samples from the Peruvian Amazon. (**B**) Proportion of oncogenic HPV types stratified by infection pattern (single vs. multiple infections). Genotypes are colored as follows: red for high-risk (HPV16, 18), purple for medium-risk (HPV45, 31, 33, 35, 52, 58), green for low-risk (HPV39, 51, 56, 59, 68), and grey for non-oncogenic types.

We assessed the distribution of HPV genotypes in general and by infection multiplicity (single vs. multiple infections). The overall distribution considering all samples (503 regular screening and 35 confirmed CIN2 + samples) can be seen in Fig. 1.

Considering all 538 women included in the study, a total of 278/538 (51.67%) tested positive for HPV. Among them, 155/278 (51.76%) cases involved single infections and 123/278 (44.24%) involved multiple infections.

Analysis of single infections (*n* = 155) revealed that up to 94/155 (60.65%) were caused by oncogenic HPV types. The most prevalent oncogenic genotypes were HPV16 (22/94, 23.40% of single infections caused by oncogenic types) and HPV52 (*n* = 16/94, 17.02%). High-risk types (HPV16 and HPV18) were identified in 25/94 (26.60%) single infections caused by oncogenic types, while medium-risk types (HPV31, 33, 35, 45, 52, and 58) accounted for 48/94 samples (51.06%), and low-risk types (HPV39, 51, 56, 59, and 68) for 21/94 cases (22.34%). Only 8 of the 155 women (5.16%) with single HPV infections reported prior HPV vaccination. None of them belonged to the group presenting histologically confirmed CIN2 + diagnosis, and only one carried a high- or medium-risk HPV type (HPV58).

Among 123 women with multiple infections, oncogenic HPV types were detected in 98/123 (79.67% of multiple HPV infections), with the most frequently detected oncogenic genotypes being HPV 16 (*n* = 25/98) and HPV 52 (*n* = 19/98). (Fig. 1B) High-risk genotypes were found in 28/98 (28.57%) multiple HPV infections presenting oncogenic types, medium-risk genotypes (excluding samples with high-risk and/or low genotypes) in 45/98 (45.92) samples, and low-risk genotypes (excluding samples with high and/or medium risk types) were detected in 25/98 women (25.51%). Only 10/98 women with multiple infections had reported being vaccinated, of whom 7/10 presented HPV types involving at least one oncogenic HPV type.

Among the 35 women with confirmed CIN2 + lesions, 27 of them showed HPV positivity. HPV16 was the most prevalent genotype (*n* = 11/27, 40.74%), followed by HPV52 (*n* = 8/27, 29.63%) and HPV31 (*n* = 3/27, 11.11%). High-risk genotypes (HPV16 and HPV18) were identified in 12/27 cases (44.44%), while medium-risk genotypes (HPV31, 33, 45, 52, and 58), in the absence of high-risk types, were detected in 8/27 cases (29.63%) and low risk types (excluding samples with high and/or medium risk types) in 7/27 samples (25.96%).

Self-reported vaccination status was available for both cohorts. Among the screening women, 42/503 (8.35%) reported prior HPV vaccination. Of these, 18/42 (42.86%) tested HPV-positive. Five out of 18 (27.79%) carried high-risk and/or medium oncogenic HPV types: 2/5 (40.00%) with multiple infections including HPV16/18/52 (with additional low-risk types), 1/5 (20.00%) with HPV33 (with additional non-oncogenic types), and 2/5 (40.00%) with HPV58 (one woman with a single infection and another with additional non-oncogenic types). The remaining infections involved low- or non-oncogenic types. Importantly, none of the 35 women with CIN2 + lesions reported previous vaccination.

### Summary of sequencing results

All single infections positive for HPV16 or HPV 52 (*n* = 36) were sequenced alongside a positive control (HPV16 plasmid) and a negative control (PCR-grade water), generating between approximately 2.3 million and 140 million trimmed reads per sample (Supplementary Table S1). The proportion of reads mapping to the HPV genome varied substantially and showed a strong negative correlation with Allplex28 Ct values for the sequenced type (Supplementary Table S1).

Of the sequenced samples (*n* = 36), 29 achieved > 90% genome coverage at a depth of ≥ 10×, and, among those, 24 also reached ≥ 100× coverage across the full viral genome. Sequencing performance was significantly associated with Ct values: both the number of reads mapped to the HPV genome and the mean coverage were strongly negatively correlated with Ct (Spearman’s ρ = − 0.98, *p* < 2.2 × 10^−16^).

### Sublineage typing results

Sublineage typing was performed for all samples with ≥ 20× coverage across the full viral genome and a minimum genome coverage threshold of 50%, resulting in 13 HPV16 positive and 17 HPV52 positive samples included in the analysis.

Among HPV16 positive samples, most clustered within sublineage A1 (11/13, 84.62%), while one sample each was assigned to D2 and D3. For HPV52, the majority of samples were classified as sublineage A1 (14/17, 82.35%), with three samples assigned to sublineage C2 (17.65%). All HPV52 sublineage assignments were supported by high bootstrap values (≥ 92). In contrast, HPV16 bootstrap support values varied more widely (51–100), with lower values typically observed in samples with a greater proportion of missing genomic positions, which likely reduced the reliability of phylogenetic placement.

### Comparison between Peruvian and Norwegian HPV16A1 and HPV52A1 strains

To explore viral diversity across time and geography, Peruvian HPV16 A1 and HPV52 A1 sequences with at least 80% genome coverage were compared to Norwegian sequences from the National Screening Program, previously published by Hesselberg Løvestad et al.^10^.

**Fig. 2:**
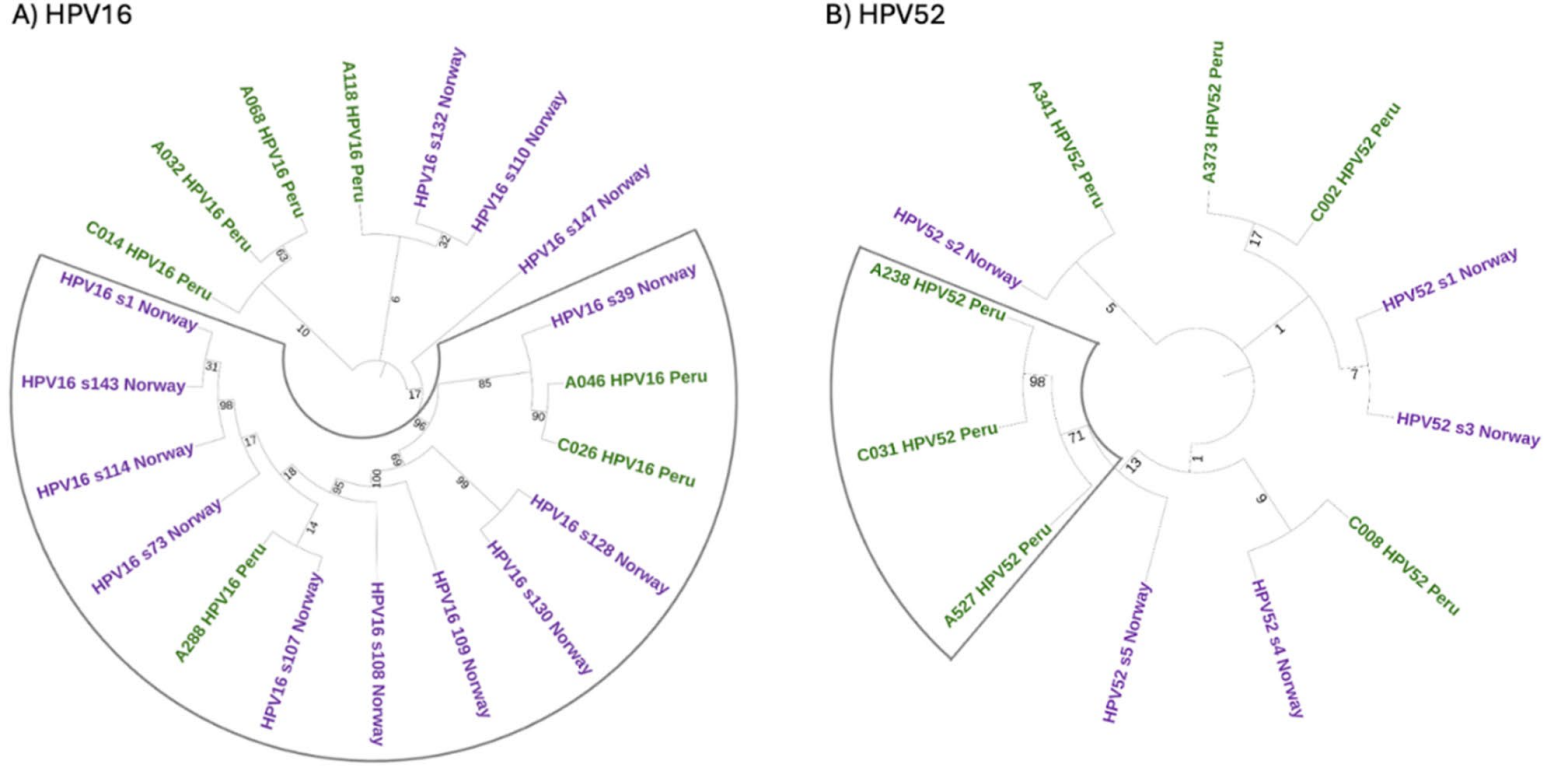
Phylogenetic comparison of HPV16 and HPV52 A1 sublineages from Peruvian and Norwegian samples. Maximum likelihood phylogenetic trees were constructed using RAxML-NG v1.2.2 under the GTR model, and 1000 bootstrap replicates^18^. Colored labels indicate country of origin (green: Peru; purple: Norway). The boxed clade marks the most strongly supported node.

The HPV16 phylogenetic tree revealed a large, well-supported clade (bootstrap = 96) comprising both Peruvian (*n* = 3) and Norwegian (*n* = 10) sequences (Fig. 2A). In contrast, the HPV52 tree exhibited minimal phylogenetic structure. All sequences were classified as sublineage A1, and most internal branches showed low bootstrap support. Aside from one clade containing three Peruvian samples (bootstrap = 71), the remaining sequences were interspersed throughout the tree without clear clustering by geographic origin or collection period (Fig. 2B).

### Mutational signatures of the intrahost variability

**Fig. 3:**
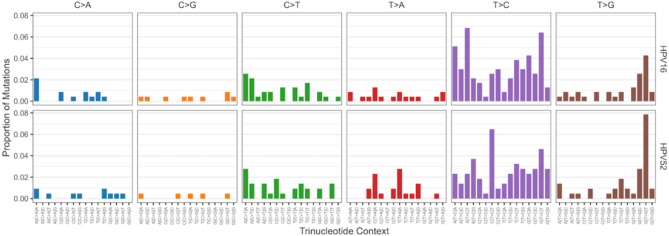
Mutation signatures of iSNVs. The bar plots show the distribution of 96 trinucleotide-based single base substitution (SBS) classes detected in HPV16 and HPV52 genomes, categorized by substitution type and context. T > C transitions were the most frequent, followed by C > T and T > G substitutions. A notable enrichment of T > G mutations within the GTT > GGT context was observed in HPV52 samples.

All detected iSNVs were classified into the 96 single base substitution (SBS) mutation classes (categories) according to trinucleotide context and substitution type (Fig. 3). In both HPV16 and HPV52, T > C substitutions predominated, followed by C > T and T > G substitutions. The relative distribution of these mutations differed between genotypes: HPV16 exhibited a broader range of T > C and C > T contexts, whereas HPV52 showed a distinct enrichment of specific T > G mutations, particularly within the GTT > GGT trinucleotide motif.

### Integrations

**Fig. 4:**
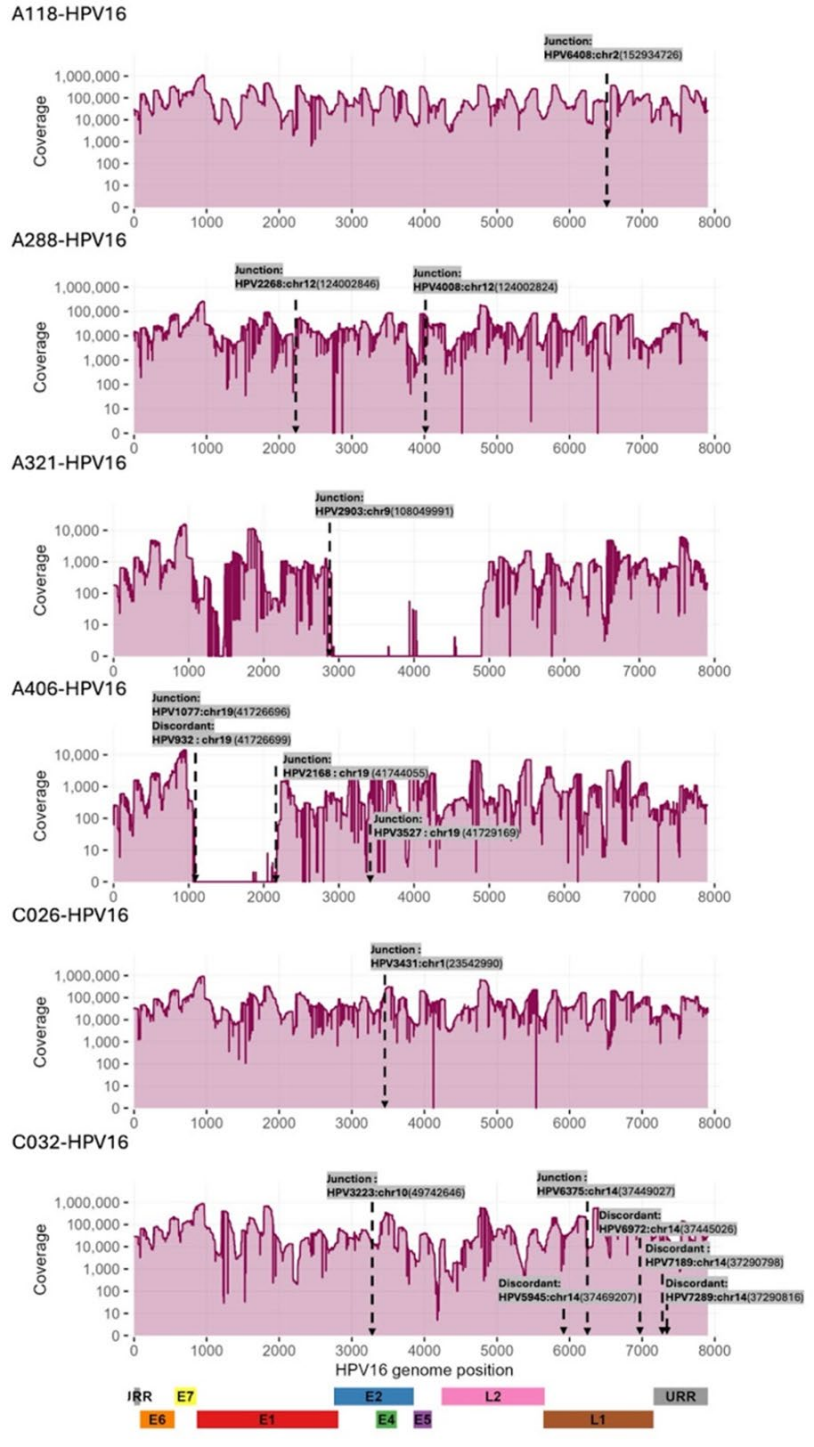
Integrations sites detected in HPV16 positive samples. The X-axis represents the full-length HPV16 genome (~ 7906 bp), while the Y-axis shows read coverage depth. Integration sites were detected by the presence of discordant and junction reads. Drops in coverage indicate potential integration breakpoints. The lower panel illustrates the genomic organization of HPV16, including early (*E6*,* E7*,* E1*,* E2*,* E4*,* E5*), late (*L1*,* L2*), and non-coding (URR) regions.

**Fig. 5:**
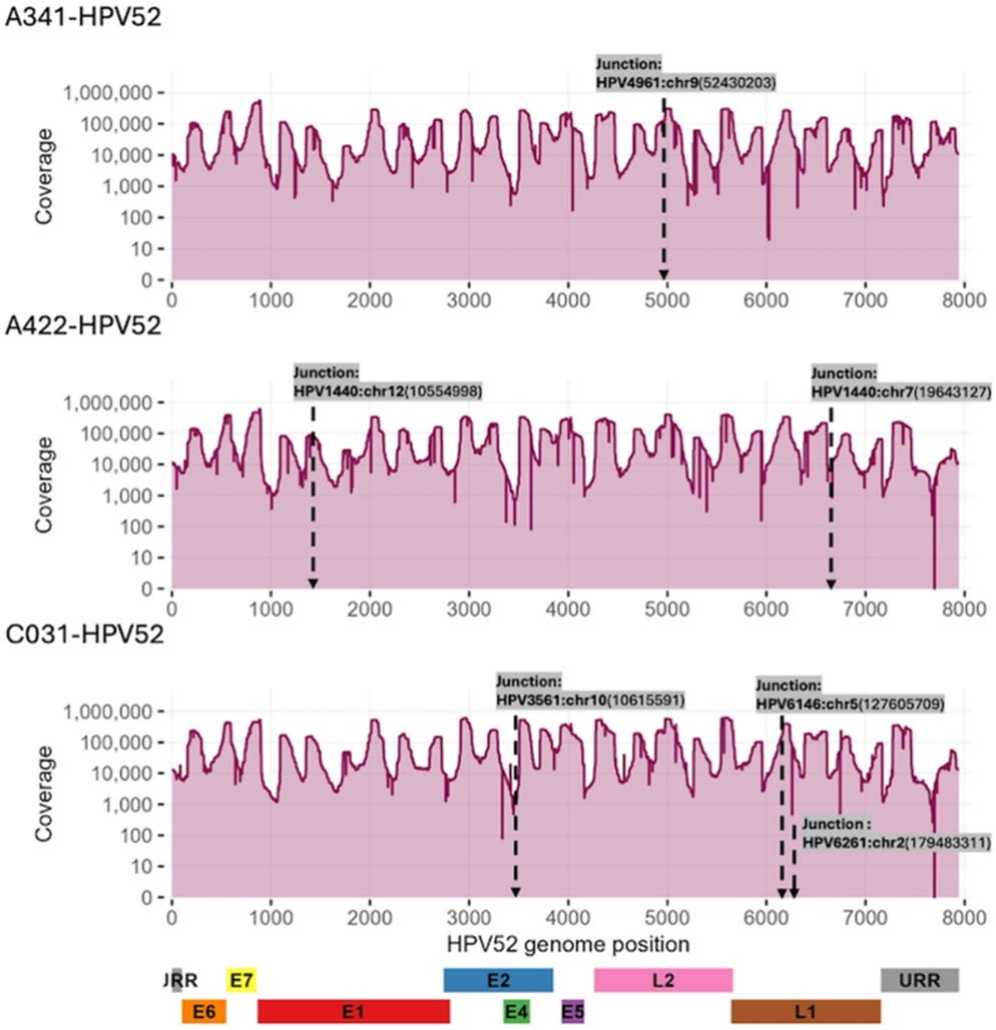
Integrations sites detected in HPV52-positive samples. The X-axis shows the HPV52 genome (~ 7961 bp) and the Y-axis displays read coverage. Integration breakpoints were detected predominantly in L1 and L2 regions. The schematic below outlines the structure of the HPV52 genome with annotated coding regions.

Integration events between the HPV genome and the human genome were detected in both HPV16 and HPV52 positive samples using a combination of discordant read pair detection and junction read analysis. In total, six HPV16 positive and three HPV52 positive samples showed evidence of high-confidence integration sites (Figs. 4 and 5). Integrations were also detected in three confirmed CIN2 + cases (C026-HPV16, C032-HPV16, and C031-HPV52). For HPV 16, integration breakpoints were observed across various regions of the viral genome, most frequently within or near the *E1*, *E2*, and *L2* genes (Fig. 4). Several samples exhibited multiple integration sites. In A321 and A406 samples, integration sites were associated with local drops in sequencing depth. Integration breakpoints varied in genomic location on the host side, involving multiple chromosomes. For HPV52, integration was also detected in diverse regions of the viral genome, mostly in *L1* and *L2* (Fig. 5). Integration breakpoints were distributed across several human chromosomes, again without apparent preference for specific genomic features.

## Discussion

This study provides the first comprehensive molecular characterization of HPV infections among women undergoing cervical screening in the Peruvian Amazon, a region burdened with some of the highest cervical cancer mortality rates in the country^1,2^.

A remarkably high prevalence of oncogenic HPV types (192/538, 35.69%) was detected among women attending screening, with over half exhibiting multiple HPV infections. These findings are consistent with previous reports from Peru’s most resource-limited regions and far exceed prevalence rates documented in urban populations^1,19^. Vaccination coverage in this study was low (42/538, 7.81%), mostly among younger women, reflecting the relatively recent introduction of HPV vaccination in Peru (2011). Among vaccinated participants, 42.9% tested HPV-positive, but the majority of infections involved low- or non-oncogenic types. Only five vaccinated women carried oncogenic HPV types, including HPV16/18/52 in multiple infections, HPV33, and HPV58. Importantly, none of the 35 women with histologically confirmed CIN2 + lesions had been vaccinated. These findings are consistent with the expected protective effect of HPV vaccination against high-grade cervical disease, while also highlighting the urgent need to improve vaccine coverage in remote regions such as Loreto.

In line with global and regional trends, HPV16 was the most common genotype identified in both single and multiple infections^9^. Supporting recent data that indicate a shift in type distribution in Latin America^1^, HPV18 was relatively rare, while HPV52 was highly prevalent, particularly among CIN2 + cases. Importantly, HPV52 is included in the nonavalent vaccine but not in the bivalent or quadrivalent formulations, underlining its oncogenic potential in this population and reinforcing the importance of implementing broader-valency vaccines in Peru.

Phylogenetic analysis revealed a predominance of A1 sublineages for both HPV16 and HPV52, in agreement with data from other Latin American populations. However, less common sublineages were also identified, including D2 and D3 for HPV16 and C2 for HPV52. These have been associated with increased oncogenicity in other regions^20,21^. Comparative phylogenetic analysis with Norwegian strains revealed tight clustering of some Peruvian HPV16 A1 variants with sequences collected more than a decade earlier in Scandinavia, suggesting that certain A1 variants have circulated globally over extended periods while still accumulating detectable sequence divergence. In contrast, HPV52 sequences showed minimal phylogenetic structure, with weak bootstrap support and no clear geographic or temporal clustering. This may reflect a slower evolutionary rate or higher sequence conservation in HPV52, though additional data are needed to confirm this.

Analysis of iSNVs revealed consistent mutational patterns across HPV16 and HPV52, dominated by T > C and C > T substitutions. These mutations are likely shaped by host deaminase activity, particularly that of the APOBEC3 enzyme family, which is known to introduce such changes in viral genomes^22–24^. The enrichment of specific T > G transversions in the GTT trinucleotide context observed in HPV52 samples is particularly notable, as such mutations are rarely reported in papillomavirus genomes. This atypical pattern may reflect genotype-specific host-virus interactions or distinct selective pressures acting on HPV52. The observed differences in mutational context may also be indicative of differential APOBEC activity across HPV genotypes and warrant further investigation.

HPV DNA integration into the human genome is a hallmark of cervical carcinogenesis^25^. Integration events were detected in nine samples, including three confirmed CIN2 + cases. Consistent with prior studies, HPV16 integration breakpoints clustered in the E1 and E2 regions of the viral genome, supporting the hypothesis that disruption of these regulatory genes promotes oncogene overexpression^25^. HPV52 integration breakpoints were more often located in *L1* and *L2*, possibly reflecting genotype-specific patterns. Integration sites were distributed across multiple human chromosomes, with no apparent enrichment for specific loci, reinforcing the notion that integration is a largely stochastic process with oncogenic potential. In two samples (A321 and A406), integration sites coincided with localized drops in sequencing depth, an observation commonly reported for HPV16 and interpreted as indicative of large deletions around integration breakpoints.

This study has several strengths. It provides valuable molecular data from a remote and low-resource region previously excluded from HPV genomic research and includes cases with confirmed high-grade lesions, strengthening clinical relevance. The application of TaME-seq enabled high-resolution analysis of viral integration and intrahost diversity, features that go beyond conventional genotyping and offer insights into viral behavior. However, limitations must be acknowledged. First, the sample size for CIN2 + lesions was modest, and sequencing performance varied with sample adequacy and viral load, as shown by the strong inverse correlation between Ct values and sequencing coverage. Second, although international phylogenetic comparisons were included, the cross-sectional design precludes inferences about transmission dynamics or viral evolution. Additionally, progression risk could not be assessed, as longitudinal follow-up data were not available.

The findings of this study have direct public health implications for the Peruvian Amazon. The high prevalence of oncogenic HPV types particularly HPV52, which is not covered by the bivalent or quadrivalent vaccines reinforces the urgent need to implement vaccination schemes using the nonavalent vaccine in regions with a high viral burden. Furthermore, the detection of viral integration in confirmed CIN2 + cases supports the use of molecular testing as a primary screening strategy over conventional cytological methods, especially in areas with limited access to clinical follow up. Implementing high-risk HPV based screening, combined with genotyping and integration surveillance, could optimize the identification of women at risk and improve cervical cancer outcomes in vulnerable settings such as Loreto.

In conclusion, this study reveals a high burden of HPV infection, including oncogenic HPV types, in women attending cervical screening in the Peruvian Amazon. The predominance of vaccine-preventable types, combined with low vaccine coverage and confirmed integration in CIN2 + cases, underscores the need to scale up HPV vaccination and strengthen molecular screening in nationwide settings. These efforts are critical for developing equitable and effective cervical cancer prevention strategies in Peru and similar high-burden regions.

## Methods

### Study population

Women aged 25 to 55 years attending cervical screening clinics at Hospital Regional de Loreto “Felipe Santiago Arriola Iglesias” and Hospital de Apoyo de Iquitos “César Garayar García”, located in the Loreto region of the Peruvian Amazon, were invited to participate in a study on the distribution of HPV genotypes for cervical HPV infection. The study was conducted between May and November 2024.

Although Peruvian cervical cancer screening guidelines recommend testing for women aged 30–49 years in accordance with World Health Organization (WHO) recommendations^8^, this study was carried out in areas with limited access to healthcare services. To address this gap and capture a broader picture of HPV circulation, all women within the approved age range (25–55 years) who attended the participating clinics, regardless of screening eligibility, were offered HPV testing. This also included accompanying women who were not initially seeking screening but agreed to participate. Exclusion criteria included women with no history of sexual activity, pregnancy, women with a history of prior hysterectomy, and those currently undergoing pharmacological or surgical treatment for cervical cancer.

To ensure the inclusion of high-grade lesions, an additional set of samples from women with histologically confirmed CIN2 + lesions was included. During the same study period (May to November 2024), all women with a clinical suspicion of CIN2 + based on cytological findings who had been referred for biopsy to the Pathology Units at the same hospitals were invited to participate. A cervical sample for HPV testing was collected immediately prior to the biopsy procedure, following written informed consent. Histological examination of the biopsies subsequently confirmed the CIN2 + diagnosis.

### Sample collection and DNA extraction

Cervical samples were collected by healthcare professionals using FLOQSwabs^®^ (Copan^®^) and stored in Copan eNAT^®^ transport and preservation medium to maintain the integrity of the genetic material during transport and storage. Genomic DNA was extracted using the STARMag 96 ProPrep system (Seegene^®^), following the manufacturer’s instructions for efficient DNA extraction. The extraction was performed using the SEEPREP 32 automated equipment (Seegene^®^), ensuring reproducibility and consistency across all processed samples.

### HPV genotyping and sample selection for variant analysis

HPV genotyping was performed using the Allplex™ HPV28 Detection Kit (Seegene^®^), which allows for the simultaneous detection of 28 HPV types, using the CFX96 Thermocycler (Biorad^®^). The manufacturer’s instructions were followed accordingly.

HPV types were classified as high-risk (HPV16, 18), medium-risk (45, 31, 33, 35, 52, 58) and low-risk (39, 51, 56, 59 and 68), based on international evidence from the global attribution analysis of HPV genotypes to invasive cervical cancer^9^. All other types were considered non-oncogenic.

### Tagmentation-assisted multiplex PCR enrichment sequencing

The most prevalent HPV genotypes detected in the study cohort were HPV16 and HPV52, both in single and multiple infections. Based on these findings, samples with single infections of HPV16 or HPV52 were selected for downstream analysis, including variant lineage determination, intrahost variability, and viral integration.

A total of 36 DNA samples, 21 HPV16 positive and 15 HPV52 positive, previously genotyped using the Allplex™ HPV28 Detection Kit (Seegene^®^), were analyzed using Tagmentation-Assisted Multiplex PCR Enrichment Sequencing (TaME-seq)^10^. Library preparation began with tagmentation using the Illumina DNA Preparation Kit (Illumina^®^), following the manufacturer’s protocol. Up to 50 ng of input DNA per sample was used in a 15 µL reaction volume. A positive control (HPV16 reference plasmid, provided by the International HPV Reference Center, www.hpvcenter.se) and a negative control (PCR-grade water) were included throughout the workflow.

Following tagmentation, multiplex PCR amplification was performed using HPV genotype-specific primers (forward and reverse reactions separated) and unique i5/i7 index adapters, as previously described^10,11^. Amplified libraries were pooled according to genotype (HPV16 or HPV52), purified using AMPure XP beads (Beckman Coulter^®^), and normalized prior to sequencing. Libraries were diluted to a final concentration of 1.8 pM and sequenced on the Illumina^®^ NextSeq 500 platform using a paired-end 151 + 151 bp run with the Mid Output reagent kit. Sequencing followed the protocols detailed in the Denature and Dilute Libraries Guide v02, NextSeq 500 System Guide v02, and NextSeq 500 Kit Reference Guide (revision F).

### Bioinformatics and downstream analysis

Bioinformatics analysis of the sequencing data was performed using the TaME-seq Snakemake workflow, which is publicly available (https://github.com/jean-marc-costanzi/TaME-seq, accessed on 2025-03-25) and has been validated and published in previous studies^10,11^. The workflow begins with quality control, including trimming of adapter sequences and filtering of low-quality reads. For alignment, two aligners were used: HISAT2^12^ for mapping the reads to the HPV genome and LAST^13^ particularly useful for detecting integration events between the HPV genome and the host genome. The final output consists of detailed statistical tables, nucleotide coverage data, and specific information regarding integration sites and intrahost viral variability.

#### Sublineage typing

Consensus sequences were generated for all samples by selecting the nucleotide with the highest read depth at each genomic position, requiring a minimum coverage of 20×. Sublineage typing was conducted separately for HPV16 and HPV52 positive samples by aligning each consensus sequence to a panel of genotype-specific sublineage reference sequences (retrieved from the PaVE database, https://pave.niaid.nih.gov/explore/variants/variant_genomes, accessed on 2025-03-25) using MAFFT v7.526^14^.

Maximum likelihood phylogenetic trees were constructed using RAxML-NG v1.2.2 under the GTR model, with 50 randomized parsimony starting trees and 1000 bootstrap replicates^15^. A sample was assigned to a specific sublineage if it clustered within a clade containing the corresponding reference sequence, with bootstrap support > 70%.

To explore viral diversity across time and geography, Peruvian HPV16 A1 and HPV52 A1 sublineage sequences were compared with Norwegian sequences obtained between 2009 and 2012 through the National Screening Program, as previously published by Hesselberg Løvestad et al.^10^.

#### Intrahost mutation detection

Intrahost single nucleotide variants (iSNV) detection was performed as previously described^10,11^. In short, raw reads were aligned to the HPV reference genome (HPV16REF, HPV52REF (retrieved from the PaVE database, https://pave.niaid.nih.gov/, accessed on 2025-03-25) using HISAT2 (v2.2.1), and nucleotide frequencies were extracted using BCFtools mpileup (v1.12)^12,16^. iSNVs were identified in a reference-independent manner as the second most abundant base at each position, with minimum ≥ 100× coverage and Phred score ≥ 30. At sites with 100–500× depth, a minimum frequency of 5% was required; for > 500× depth, a 1% threshold was applied. Only samples with ≥ 100× mean sequencing depth were included.

All detected iSNVs were classified into the 96 single base substitution (SBS) mutational signatures based on trinucleotide context. Counts were converted to proportions and visualized per HPV type to explore mutational patterns.

#### HPV-human chromosome integration detection

Viral integration analysis followed a previously described approach^10,11^. Potential integration sites were identified by detecting discordant read pairs in the HISAT2 alignment, where one read mapped to the human genome and its mate to HPV. Sites were recorded if at least two such human-mapped reads had unique start or end positions. HISAT2-unmapped reads were re-aligned with LAST to detect junction reads, and integration breakpoints were recorded if they were supported by ≥ 3 junction reads with unique coordinates. All candidate sites were visually inspected in IGV Web App (v2.2.7)^17^. Integrations supported only by split reads, repetitive regions, or likely trimming artefacts were excluded.

## Supplementary Information

Below is the link to the electronic supplementary material.

Supplementary Material 1

## Data Availability

The data that support the findings of this study are available on request from the corresponding author. The data are not publicly available due to privacy or ethical restrictions. All code used for processing and analysis of sequencing data is publicly available at the TaME-seq GitHub repository: [https://github.com/sinanugur/TaME-seq].
