# Supplementary material for: Genotype distribution and molecular characterization of HPV in the Peruvian amazon: insights into prevalence, lineage diversity, and viral integration
Source: Sci Rep. 2025 Sep 15;15:32535. doi: 10.1038/s41598-025-18455-3 (PMC12436591; doi:10.1038/s41598-025-18455-3)
Supplement: Supplementary file 1 — Supplementary Material 1 [file 41598_2025_18455_MOESM1_ESM.pdf]

Supplementary Table S1: Summary of sequencing and mapping metrics for single HPV16- or HPV52-positive samples.

Sample ID	Number of trimmed reads	Number of reads mapping to		Coverage			% of genome covered with sequencing depth greater than			Allplex28 Ct value of the sequenced type
		Human genome (hg38)	Targeted HPV	Mean	Min	Max	10x	50x	100x	
A118-HPV16	14015006	162295	14438950	123235,2	635	1087185	100 %	100 %	100 %	14,47
A068-HPV16	8772174	3013887	5756138	66646,5	1	603079	100 %	100 %	100 %	21,98
C026-HPV16	9728680	2277082	7616909	85923,7	0	926005	100 %	100 %	100 %	19,88
C032-HPV16	11619208	690110	10140316	94214,2	5	884309	100 %	100 %	99 %	17,87
A032-HPV16	5341820	4375920	850905	9808,0	0	90642	100 %	100 %	100 %	24,86
A288-HPV16	6740342	4026327	2644369	30561,2	0	260092	100 %	100 %	100 %	24,02
C014-HPV16	4928282	4288379	504149	5794,6	0	67063	100 %	100 %	99 %	25,37
A046-HPV16	3735352	3498574	100745	1122,1	0	14236	85 %	83 %	79 %	28,67
A303-HPV16	1881374	1785331	24791	288,2	0	4375	79 %	57 %	42 %	30,65
A321-HPV16	3622816	3398123	89792	1023,8	0	15621	73 %	69 %	66 %	28,08
A204-HPV16	2646708	2514242	11903	133,5	0	1410	70 %	51 %	44 %	28,64
A433-HPV16	4785500	4592627	5831	64,6	0	1461	61 %	29 %	19 %	31,09
A526-HPV16	3936346	3820157	1076	14,9	0	457	17 %	8 %	3 %	34,48
A117-HPV16	4366794	4206045	386	7,5	0	258	11 %	3 %	2 %	38,53
A125-HPV16	4652178	4524173	74	4,9	0	127	9 %	3 %	1 %	38,52
A120-HPV16	989004	958235	7	0,7	0	17	3 %	0 %	0 %	40,02
C022-HPV16	2378674	2252095	3	0,1	0	31	2 %	0 %	0 %	39,97
A162-HPV16	4602272	4465237	22	1,1	0	168	1 %	0 %	0 %	36,3
A295-HPV16	3549780	3402192	0	0	0	0	0	0	0	41,26
A334-HPV16	5478100	5285168	0	0	0	0	0	0	0	41,61
A335-HPV16	2603624	2473749	0	0	0	0	0	0	0	40,41
A341-HPV52	6589998	319406	6532278	65806,6	19	568426	100 %	100 %	100 %	19,29
C002-HPV52	8090650	219422	8111290	81656,1	13	555127	100 %	100 %	100 %	18,43
A238-HPV52	6457436	444959	6163297	64185,9	0	377216	100 %	100 %	100 %	21,09
A414-HPV52	9753606	295498	9192463	92251,5	0	597820	100 %	100 %	100 %	17,86
A422-HPV52	8994614	292917	8495661	85766,4	1	617688	100 %	100 %	100 %	18,22
C031-HPV52	12563058	138664	12293131	112895,4	1	616558	100 %	100 %	100 %	17,29
C008-HPV52	834312	652488	271491	2996,6	0	19200	100 %	99 %	98 %	24,75
A373-HPV52	3963522	3708589	286145	2974,1	0	33042	98 %	94 %	87 %	25,35
A527-HPV52	2835602	2845917	139644	1613,5	0	9051	94 %	85 %	78 %	27,57
A453-HPV52	3866382	3980398	38812	439,2	0	4342	86 %	65 %	48 %	32,44
C036-HPV52	963334	974397	24797	275,1	0	3313	79 %	58 %	50 %	28,94
A429-HPV52	2768918	2918987	6156	71,1	0	1178	37 %	19 %	16 %	34,53
A523-HPV52	2807840	2836219	4112	44,0	0	1441	36 %	18 %	9 %	35,03
A151-HPV52	3669852	3370401	2842	31,2	0	720	19 %	14 %	8 %	36,22
C004-HPV52	6023656	6203866	0	0	0	0	0	0	0	38,24

Each sample underwent TaME-seq analysis, including one HPV16 plasmid (positive control) and one PCR-grade water sample (negative control). The table provides, per sample: number of trimmed reads, number of reads mapping to the human genome and to the HPV type targeted (HPV targeted type can be seen in Sample ID name), sequencing coverage (average, minimum, and maximum), proportion of genome covered at >10×, >50×, and >100× depth, and Allplex™ HPV28 Ct values for the respective HPV type. A negative correlation was observed between Ct values and percentage of HPV genome coverage.
